# Traumatic xylophagia leading to foreign body removal and tracheostomy in the setting of postpartum psychosis

**DOI:** 10.1093/jscr/rjab467

**Published:** 2021-12-11

**Authors:** Brady J Anderson, David Z Allen, Sean P McKee, Garren Low, Sancak Yuksel

**Affiliations:** Department of Otorhinolaryngology–Head and Neck Surgery, McGovern Medical School at the University of Texas Health Science Center, Houston, TX, USA; Department of Otorhinolaryngology–Head and Neck Surgery, McGovern Medical School at the University of Texas Health Science Center, Houston, TX, USA; Department of Otorhinolaryngology–Head and Neck Surgery, McGovern Medical School at the University of Texas Health Science Center, Houston, TX, USA; Department of Otorhinolaryngology–Head and Neck Surgery, McGovern Medical School at the University of Texas Health Science Center, Houston, TX, USA; Department of Otorhinolaryngology–Head and Neck Surgery, McGovern Medical School at the University of Texas Health Science Center, Houston, TX, USA

## Abstract

Postpartum psychosis (PPP) is a severe mood disorder following childbirth that rarely leads to injurious or suicidal behavior. This report illustrates otolaryngologic intervention for pharyngeal laceration and airway instability following traumatic foreign body ingestion in the setting of PPP.

A 25-year-old woman with PPP presented with hemoptysis after attempting suicide by traumatically forcing tree branches into her oropharynx. Imaging revealed pneumomediastinum, and flexible laryngoscopy and esophagoscopy showed a large foreign body (tree branch) extending from the hypopharynx to the gastroesophageal junction. She was taken to the operating room for direct microlaryngoscopy, bronchoscopy and esophagoscopy with removal of the 25-cm tree branch. Panendoscopy revealed a mucosal laceration at the cricopharyngeus with supraglottic and hypopharyngeal edema but no injury to the larynx. Due to airway concerns, a cuffed tracheostomy was placed along with a gastrostomy tube for feeding access. She tolerated her postoperative course with successful decannulation and oral feeding prior to discharge.

## INTRODUCTION

Postpartum psychosis (PPP) is defined as severe mood disturbance following childbirth, possibly with severe and injurious behavior [1]. Hospitalization expedites treatment and ensures patient safety, as cases of suicide and filicide have been reported [2]. Given its tenuity, providers should understand its presentation and initial management, including psychiatric consultation. Only one case of foreign body ingestion (FBI) has been reported in PPP, describing ingestion of a safety pin, nut and bolt at the behest of command auditory hallucinations [3].

Unlike small objects which frequently pass spontaneously, ingestion of large or sharp foreign bodies (FB) may lead to life-threatening complications, including airway obstruction, hemorrhage and esophageal perforation with mediastinitis [4, 5]. In such cases, endoscopic removal or surgical management are critical for airway stability and prevention of further internal trauma [4, 5]. We report the successful management of a patient with pharyngeal injury, hemorrhage and airway instability following traumatic xylophagia in the setting of PPP. This report highlights the urgent nature of PPP and the importance of prompt intervention for large FB ingestion.

## CASE REPORT

A 25-year-old woman, 3-month postpartum, presented to an outside hospital after attempting suicide by forcing tree branches into her oropharynx. She presented with muffled voice and active hematemesis, with no oral source of bleeding visualized. Due to airway concerns, she was intubated with a 7.5 cuffed endotracheal tube. Computed tomography revealed cervical subcutaneous emphysema and pneumomediastinum, raising concern for esophageal perforation. The patient was given broad-spectrum antibiotics and transferred for higher level of care.

In the receiving ED, cervical crepitus was palpable and bedside direct laryngoscopy, esophagogastroduodenoscopy, and flexible bronchoscopy revealed a tree branch extending from the proximal esophagus to the gastroesophageal junction (GEJ). A posterior pharyngeal laceration was noted at the level of the cricopharyngeus, but no FB was observed within the trachea or bronchi. The patient was taken emergently to the operating room (OR) for panendoscopy of the larynx, bronchus and esophagus, with FB removal via rigid esophagoscopy.

**Figure 1:**
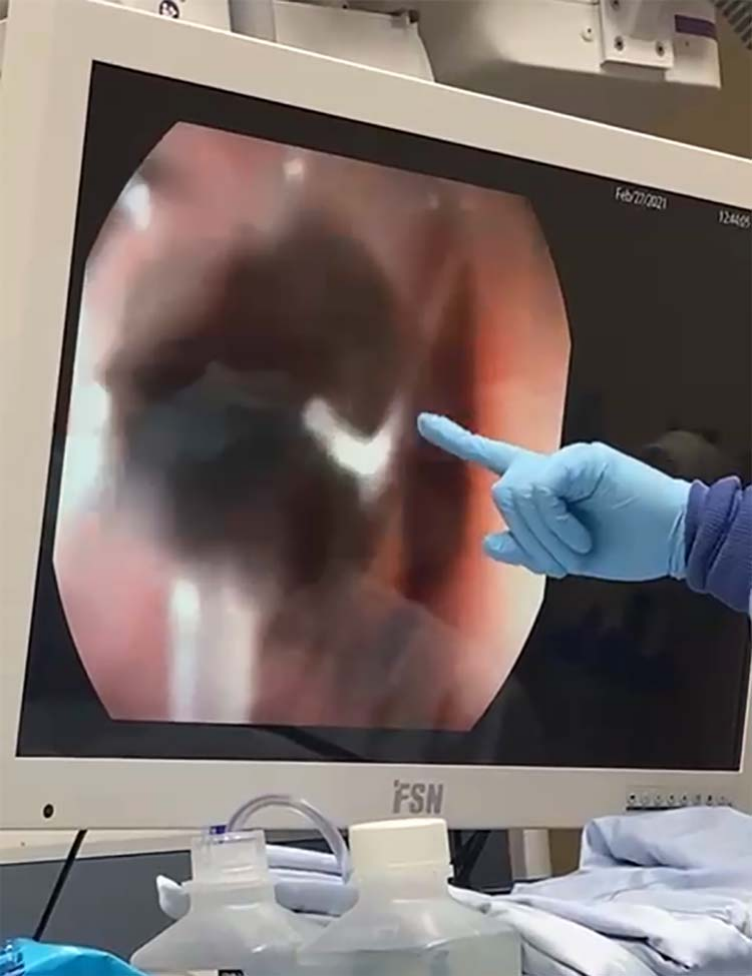
The FB (tree branch) extending from the proximal esophagus to the GEJ.

**Figure 2:**
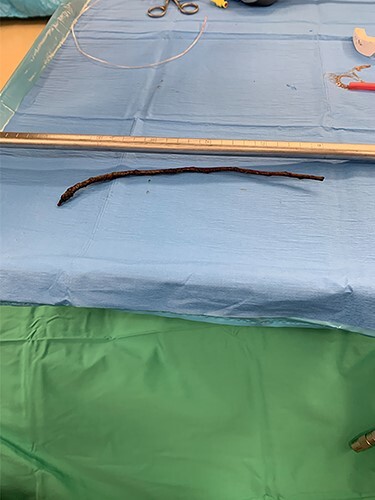
The 25-cm tree branch following removal.

In the OR, direct microlaryngoscopy revealed the aforementioned hypopharyngeal laceration as well as significant supraglottic ecchymosis, edema and partial airway obstruction. However, the vocal folds and distal trachea were atraumatic. A rigid esophagoscope was introduced and advanced to the GEJ, and the 25-cm tree branch was removed (Figs 1 and 2). The esophagus was visualized to be intact. The hypopharyngeal laceration appeared well approximated and primary closure was deferred. Due to the supraglottic edema, a surgical tracheostomy was performed, and a 6.0 cuffed Shiley tracheostomy tube was placed. Then, to facilitate long-term tube-feeding, a percutaneous endoscopic gastrotomy tube was placed by trauma surgery.

Postoperatively, the patient received steroids and antibiotics and was ventilated in the intensive care unit for 1 day. She was eventually transitioned to trach collar, and on postoperative day (POD) three was changed to a cuffless trach and transferred to the floor. Serial radiographs demonstrated resolving pneumomediastinum without signs of esophageal perforation. On POD 13, a gastrograffin study demonstrated no esophageal leak but did raise concern for aspiration. A barium study was subsequently performed by speech–language pathology, revealing minor swallowing deficits without aspiration. After education on safe swallowing, the patient was decannulated and transitioned to regular diet.

Postoperatively, the patient was evaluated and treated by inpatient psychiatry. She reported worsening insomnia, anxiety and auditory hallucinations since giving birth 3 months earlier, culminating in commands to attempt suicide by tree branch ingestion. Psychiatry diagnosed her with late-onset PPP and prescribed olanzapine and valproic acid with haloperidol for agitation. Inpatient psychiatric transfer was initially recommended but not completed during her admission.

By POD 24 she had no hallucinations or suicidal ideation and was discharged home with outpatient otolaryngology and psychiatry appointments. At her 2-week otolaryngology follow-up in clinic, she reported improved swallowing and phonation and was instructed to return as needed.

## DISCUSSION

PPP has an incidence of 0.1–0.2% in postpartum patients, with proposed mechanisms including hormonal changes, circadian rhythm disruption and immunologic and genetic factors [1]. Symptoms may include depression and anxiety, but disorganized thought, delusions, mania or hallucinations distinguish PPP from the postpartum blues [2]. PPP usually presents immediately postpartum, but late-onset cases have been reported [6].

FBI is typically encountered in children [4, 7]. In adults, intentional FBI may be associated with psychiatric conditions [4, 8, 9]. Notable cases requiring removal include a 17-cm wrench [10] and 12-cm metal spring [11]. In the one case describing FBI in the setting of PPP, no endoscopic or surgical intervention was reported, likely due to the objects’ small, blunt nature [3].

While small objects may pass uneventfully, sharp or long (>6 cm) objects require removal due to increased rates of gastrointestinal perforation [4]. This patient’s mucronate, 25-cm tree branch fulfilled both criteria, and prompt removal prevented damage beyond the posterior hypopharyngeal laceration. Esophageal perforation and large pharyngeal injuries necessitate surgical repair, but minor pharyngeal injuries may heal with antibiotics and fasting [12]. In addition, injuries above the arytenoid cartilages have shown lower rates of infectious and noninfectious complications [13]. This patient’s supra-arytenoid, shallow mucosal injury was therefore allowed to heal by secondary intention.

Tracheostomy is rarely performed for FBI, typically for alternate access when transoral removal is unattainable [14]. In contrast, upper airway obstruction is a known indication for tracheostomy [15]. This patient’s hypopharyngeal laceration, pneumomediastinum and significant supraglottic edema required temporary tracheostomy placement and mechanical ventilation, but ventilatory weaning and capping trials were performed as soon as was determined safe. In addition, attentive care by a multidisciplinary team of surgeons, psychiatrists, rehab therapists and speech pathologists facilitated this patient’s speedy, uneventful recovery, consistent with recommendations for treating FBI [8].

## CONCLUSIONS

PPP may lead to serious self-harm, as in this case of attempted suicide by traumatic xylophagia. Multidisciplinary treatment with early hospitalization and psychiatric consultation is critical for effective, timely recovery. Ingested objects that are large or sharp should be removed emergently. We managed this complicated FBI with panendoscopy, removal and tracheotomy for airway management.

## CONFLICT OF INTEREST STATEMENT

None declared.
